# Statin-Induced Coenzyme Q Deficiency Induces Metabolic Reprogramming in Astrocytes

**DOI:** 10.3390/antiox15060725

**Published:** 2026-06-07

**Authors:** Krzysztof Wojcicki, Lukasz Galganski, Adrianna Budzinska, Grzegorz Figura, Wieslawa Jarmuszkiewicz

**Affiliations:** Laboratory of Mitochondrial Biochemistry, Department of Bioenergetics, Faculty of Biology, Adam Mickiewicz University, Uniwersytetu Poznanskiego 6, 61-614 Poznan, Poland; krzysztof.wojcicki@amu.edu.pl (K.W.); lukasz.galganski@amu.edu.pl (L.G.); adrianna.budzinska@amu.edu.pl (A.B.); grzegorz.figura@amu.edu.pl (G.F.)

**Keywords:** astrocytes, atorvastatin, simvastatin, CoQ deficiency, mitochondrial function, oxidative stress

## Abstract

Statins are commonly used cholesterol-lowering drugs, but their effects on astrocyte oxidative metabolism are poorly understood. To investigate this, rat astrocytes were exposed to 200 nM atorvastatin or simvastatin for 6 days and then assessed for changes in coenzyme Q (CoQ) homeostasis, mitochondrial function, and energy metabolism. Both statins comparably decreased cellular CoQ9 and CoQ10 levels (~35%), with greater losses of their reduced antioxidant forms (60–75%). Lower intracellular and mitochondrial levels of reactive oxygen species (ROS) were accompanied by the upregulation of nuclear factor erythroid 2-related factor 2 (NRF2)-dependent antioxidant pathways (superoxide dismutase 1 and glutathione reductase) and metabolic stress response factors, including hypoxia-inducible factor 1-alpha (HIF1α) and brain-derived neurotrophic factor (BDNF). Both statins promoted glycolytic reprogramming, mitochondrial fission, and biogenesis while impairing oxidative phosphorylation, as evidenced by reduced ATP-linked respiration, increased proton leak, and lower ATP levels. These findings suggest that statin-treated astrocytes adapt by prioritizing redox homeostasis over ATP production. CoQ10 supplementation increased cellular CoQ10 levels and restored ATP levels without further decreasing ROS, suggesting that its primary benefit is bioenergetic support, not additional antioxidant protection. Overall, statin-induced CoQ deficiency induces adaptive metabolic remodeling of astrocytes, while CoQ10 supplementation may help maintain energy metabolism under these conditions.

## 1. Introduction

Statins are commonly prescribed, lipid-lowering medications. They primarily act by inhibiting 3-hydroxy-3-methylglutaryl-coenzyme A reductase (HMG-CoA reductase), the rate-limiting enzyme in the mevalonate pathway [1,2,3]. In addition to their well-documented role in lowering blood cholesterol levels and cardiovascular risk, statins exert various pleiotropic effects, including modulation of inflammation, oxidative stress, and cellular metabolism [4,5]. Increasing evidence suggests that these effects may extend to the central nervous system (CNS), as several statins, including atorvastatin and simvastatin, can cross the blood–brain barrier and affect brain function [6].

In addition to cholesterol synthesis, the mevalonate pathway produces several essential isoprenoid intermediates, including farnesyl pyrophosphate and geranylgeranyl pyrophosphate, which are required for posttranslational prenylation of small GTP-binding proteins involved in mitochondrial dynamics, intracellular transport, and cell survival signaling [1,2,3]. Therefore, statins’ inhibition of this pathway may alter mitochondrial function by interfering with protein prenylation, impairing mitochondrial biogenesis, and modifying the efficiency of oxidative phosphorylation [7,8]. Statins also reduce the synthesis of coenzyme Q (CoQ), an important intracellular antioxidant and a key component of the mitochondrial electron transport chain, which may affect electron transfer and the production of ATP and reactive oxygen species (ROS) [9,10].

Astrocytes are the most abundant glial cells in the brain and play a crucial role in maintaining neuronal homeostasis. They are essential regulators of brain energy metabolism, providing neurons with metabolic substrates, controlling redox balance, and supporting synaptic transmission [11,12]. Astrocytes rely primarily on aerobic metabolism, and mitochondrial oxidative phosphorylation is crucial for maintaining their physiological functions. Therefore, changes in mitochondrial integrity or efficiency induced by statin inhibition of the mevalonate pathway may require metabolic adaptations in astrocytes to maintain energy homeostasis.

Besides lowering cholesterol levels, statins have been shown to exert multiple direct effects on astrocytes [13]. In cultured rat astrocytes, lipophilic statins such as atorvastatin and simvastatin induce time- and dose-dependent stellation and subsequent apoptosis [14]. This effect is reversed by mevalonate and geranylgeranyl pyrophosphate, suggesting that isoprenoid depletion may lead to astroglial toxicity. Evidence from in vivo and ex vivo models of CNS injury indicates that simvastatin attenuates astrogliosis and reactive activation of astrocytes, reduces their expression of proinflammatory cytokines, and suppresses nuclear factor kappa B (NF-κB) signaling, in part by modulating caveolin 1 (CAV1)/epidermal growth factor receptor (EGFR) signaling in lipid rafts and inhibiting neurotoxic type A1 polarization of astrocytes [15,16,17,18]. In cultured cortical astrocytes, simvastatin significantly attenuates tissue-type plasminogen activator (PLAT/tPA)-induced upregulation of matrix metalloproteinase 9 (MMP9) by inhibiting the Rho/Rho-associated coiled-coil containing protein kinase (ROCK) signaling pathway. This mechanism may contribute to the neurovascular protective effects of statins in ischemic injury models [19].

However, relatively little is known about the effects of statins on oxidative metabolism, ROS production, and CoQ homeostasis in astrocytes. Given astrocytes’ key role in brain energy homeostasis, understanding statin-induced metabolic changes in astrocytes is crucial for assessing their potential impact on the proper functioning of the nervous system.

Therefore, this study examined the effects of chronic, 6-day exposure to atorvastatin or simvastatin on oxidative metabolism and mitochondrial function in the rat astrocytes. It assessed key parameters of oxidative energy metabolism, including respiratory function, ATP levels, CoQ content, and ROS production, as well as cell viability, cytoprotective responses, and mitochondrial turnover to determine whether statins induce adaptive metabolic responses in astrocytes. It also examined the effect of coenzyme Q10 (CoQ10) supplementation on ROS production and ATP levels in statin-treated cells.

## 2. Materials and Methods

### 2.1. Chemicals

All reagents used in this study, including atorvastatin (cat. no. 1044516) and simvastatin (cat. no. 1612700), were obtained from Sigma-Aldrich (St. Louis, MO, USA). The antibodies were obtained from the sources listed in Section 2.6. Atorvastatin was prepared as a stock solution in methanol. Before use, simvastatin was converted to the active hydroxyacid form by alkaline hydrolysis of the lactone ring: the simvastatin solution was incubated at 60 °C for ~2 h, then adjusted to pH 7.2 with hydrochloric acid.

### 2.2. Cell Culture and Preparation of Cell Fractions

**Figure 1:**
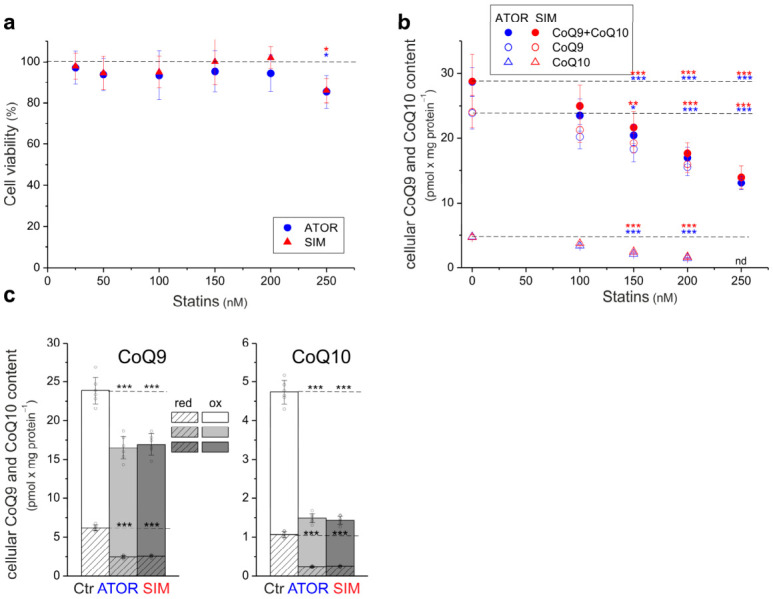
Effects of increasing concentration of statins on the (**a**) viability and (**b**) cellular CoQ9 and CoQ10 content of astrocytes (CTX TNA2). (**c**) Effects of 200 nM statins on the reduced (red), oxidized (ox), and total (red + ox) pools of CoQ9 and CoQ10. Total CoQ content (CoQ_ox_ + CoQ_red_) is shown in b. The data shown are the mean ± SD across six replicates, compared using one-way ANOVA. Significance: *, *p* < 0.05; **, *p* < 0.01; ***, *p* < 0.001 vs. control astrocytes (dashed lines). Abbreviations: ATOR, atorvastatin; nd, not detectable; SIM, simvastatin.

The experiments used the astrocyte cell line CTX TNA2 (cat. no. CRL-2006; ATCC, Manassas, VA, USA), originally derived from primary astrocytes cultured from the frontal cortex of 1-day-old rats. Cells were cultured in Dulbecco’s modified Eagle’s medium (low glucose, 1 g/L) supplemented with 10% fetal bovine serum (FBS), 2% L-glutamine, and 1% penicillin-streptomycin. Cultures were maintained at 37 °C in a humidified atmosphere containing 5% CO_2_. Cells were cultured under control conditions or exposed to 200 nM atorvastatin or simvastatin (except for the experiments described in Figure 1). Statins were added on the day of passage at the indicated final concentration. In the CoQ10 supplementation experiments, 3.4 µM CoQ10 was added to astrocytes when indicated. This concentration was chosen because it corresponds to the plasma concentration observed in individuals taking CoQ10 supplementation [20]. Cells between passages 4 and 10 were seeded into 140 mm culture dishes and maintained for 6 days until they reached 90–100% confluence, then harvested for analysis.

After 6 days of culturing, cells from the control and statin-treated groups were detached using trypsin–ethylenediaminetetraacetic acid. Next, the collected cells were washed sequentially with phosphate-buffered saline (PBS) containing 10% FBS, then with 5% FBS in PBS, and finally with PBS alone. After each washing step, the samples were centrifuged at 1200× *g* for 10 min at 4 °C. The resulting cell pellets were resuspended in ice-cold PBS at a ratio of 1 g of cells per 2 mL of buffer and stored on ice. Cell culture efficiency was comparable between the control and statin-treated groups, with an average yield of approximately 1 g of cells from seven culture dishes when seeded at identical densities.

To obtain cytoplasmic fractions for enzyme activity and ATP assays, cells were homogenized in PBS using a Polytron homogenizer (model no. T 18; IKA-Werke GmbH & Co. KG, Staufen, Germany) with ten 5 s cycles. The resulting homogenates were centrifuged at 1200× *g* for 10 min at 4 °C to remove intact cells and cellular debris.

### 2.3. Cell Viability

Cell viability was assessed using the MTT Cell Growth Assay Kit (Sigma-Aldrich, St. Louis, MO, USA). Cells were incubated with 3-(4,5-dimethylthiazol-2-yl)-2,5-diphenyltetrazolium bromide (MTT), which is metabolically reduced by mitochondrial dehydrogenases in living cells to form insoluble, purple formazan crystals. After incubation, the formazan product was dissolved in isopropanol, and absorbance was measured at 540–570 nm using a Spark microplate reader (Tecan Group Ltd., Männedorf, Switzerland). The measured absorbance intensity was proportional to the number of metabolically active cells.

### 2.4. Cellular CoQ Levels

Coenzyme Q9 (CoQ9) and CoQ10 concentrations in cells were determined by high-performance liquid chromatography (ÄKTA Pure; GE Healthcare, Chicago, IL, USA) using a LiChrosorb RP-18 column (10 µm; Hichrom, Theale, UK) according to established analytical protocols [21,22]. CoQ species were detected spectrophotometrically at 275 nm for the reduced forms and 290 nm for the oxidized forms. CoQ9 and CoQ10 were quantified and calibrated using commercially available standards.

Cell samples were analyzed under completely oxidizing conditions, omitting respiratory substrates that reduce CoQ. Before extraction, 15 mg of cells were incubated in 3 mL of PBS for 10 min with gentle mixing.

### 2.5. ROS Determination

Total cellular ROS production was quantified using 5 μM 5-(i-6)-chloromethyl-2′,7′-dichlorodihydrofluorescein in acetyldiacetane (CM-H_2_DCFDA) (Thermo Fisher Scientific, Waltham, MA, USA), while mitochondrial superoxide generation was assessed using 5 μM MitoSOX^TM^ Red (Thermo Fisher Scientific, Waltham, MA, USA). Cells (50 μg protein/mL) were incubated with the appropriate fluorescent probes in PBS supplemented with respiratory substrates glucose (5.5 mM) and pyruvate (5 mM) for 10 min at 37 °C. After incubation, cells were washed twice with PBS, centrifuged at 1200× *g* for 10 min at 4 °C, and resuspended in PBS to a final concentration of 50 μg protein/mL. Fluorescence was measured in 96-well plates using a Spark microplate reader (Tecan Group Ltd., Männedorf, Switzerland) at excitation/emission wavelengths of 510/595 nm for MitoSOX^TM^ Red and 495/522 nm for CM-H_2_DCFDA.

### 2.6. Protein Immunodetection

Cells were lysed in RIPA buffer containing 150 mM NaCl, 50 mM Tris-HCl (pH 8.0), 1% Triton X-100, 0.5% sodium deoxycholate, and 0.1% sodium dodecyl sulfate (SDS). Protein samples were separated on 8–12% SDS-polyacrylamide gels. The Spectra^TM^ Multicolor Broad Range Protein Ladder (Thermo Fisher Scientific, Waltham, MA, USA) was used as a molecular weight marker. The following primary antibodies were used to detect specific proteins (obtained from Abcam [Cambridge, UK] unless otherwise stated): brain-derived neurotrophic factor (BDNF, 14 kDa, ab226843); citrate synthase (CS, 46 kDa, ab96600); dynamin related protein 1 (DRP1, 95 kDa, ABT155; Sigma-Aldrich, St. Louis, MO, USA); glutathione reductase (GR, 60 kDa, ab128933); mitochondrial cleavage factor (MFF, 37 kDa, ab129075), mitofusin 1/2 (MFN1/2, 80 kDa, ab57602); nuclear factor erythroid 2-related factor/ NFE2-like bZIP transcription factor 2 (NRF2/NFE2L2, 95 kDa, ab137550); peroxisome proliferator-activated receptor γ coactivator 1α (PGC1α, 92 kDa, ab54481); superoxidase dismutase 1 (SOD1, 18 kDa, ab13498); transcription factor A, mitochondrial (TFAM, 25 kDa, ab131607); voltage-dependent anion-selective channel protein 1 (VDAC1, 35 kDa, ab14734); mitochondrial dynamin-like GTPase (OPA1, 100 and 80 kDa, BDB612607; Thermo Fisher Scientific, Waltham, MA, USA); hypoxia-inducible factor 1-alpha (HIF1α, 115 kDa, PA5-85494); lactate dehydrogenase (LDH, 35 kDa, PA5-27406); phospho-dynamin related protein 1 (Ser616) (Phospho-DRP1, 95 kDa, PA5-64821 (Thermo Fisher Scientific, Waltham, MA, USA); extracellular signal-regulated protein kinase (ERK1/2, 42/44 kDa, #4695); phospho-ERK1/2 (Thr202/Tyr204), (42/44 kDa, #9101) (Cell Signaling Technology, Danvers, MA, USA), cytochrome *c* oxidase subunit 2 (COXII, 24 kDa, orb411834, Biorbyt, Cambridge, UK); and hexokinase I (HK I, 120 kDa, sc-80978) Santa Cruz Biotechnology (Dallas, TX, USA). Actin (42 kDa, CP01; Merck, Darmstadt, Germany) was used for data normalization. Blots were cut prior to antibody incubation when necessary. Uncropped images with corresponding protein loading controls are shown in Supplementary Figures S1–S4. The protein bands were analyzed densitometrically using ImageJ 1.x software (U.S. National Institutes of Health, Bethesda, MD, USA).

### 2.7. Enzyme Activity

Enzyme activity in cytoplasmic fractions was analyzed using a Shimadzu UV-1620 spectrophotometer (Shimadzu Corporation, Kyoto, Japan) at 37 °C under constant stirring. Citrate synthase (CS) activity was measured by detecting the formation of 5,5′-dithiobis(2-nitrobenzoic acid)-coenzyme A (DTNB-CoA) at 412 nm. The reaction mixture contained 100 µM oxaloacetate, 100 µM acetyl-CoA, 100 µM DTNB, 0.1% Triton X-100, 100 mM Tris–HCl buffer (pH 8.0), and 100 µg/mL of cytoplasmic protein.

Lactate dehydrogenase (LDH) activity was determined by monitoring the oxidation of NADH (200 µM) at 340 nm. The reaction mixture contained 20 mM pyruvate, 50 mM Tris–HCl buffer (pH 7.3), and 100 µg/mL of cytoplasmic protein.

### 2.8. Cellular ATP Content

Intracellular ATP concentration in cells was quantified using the Luminescent ATP Detection Assay Kit (ab113849, Abcam, Cambridge, UK). After cell lysis, luciferase and luciferin reagents were added, and luminescence was recorded using a Spark multimode plate reader (Tecan Group Ltd., Männedorf, Switzerland).

### 2.9. Cellular Respiration

The cellular oxygen consumption rate (OCR) was assessed polarographically at 37 °C using a Clark-type oxygen electrode (Hansatech Instruments Ltd., Pentney, UK). Measurements were performed in 0.6 mL of a reaction medium containing 0.8 mM MgSO_4_, 5.4 mM KCl, 110 mM NaCl, 1.1 mM NaH_2_PO_4_, 44 mM NaHCO_3_, and 10 mM Na/Na buffer (pH 7.5). Cells were used at a final protein concentration of 3 mg/mL. Basal respiration was assessed in the presence of various substrates, including 5.5 mM glucose, 5 mM pyruvate, 4 mM glutamine, or a mixture thereof. To determine proton leak (OCR unrelated to ATP), ATP synthase activity was inhibited by adding oligomycin (1 µg/mL). The ATP-linked OCR was calculated as the difference between the basal OCR and proton leak. The maximum OCR was then achieved by adding the uncoupler carbonylcyanide-*p*-trifluoromethoxyphenylhydrazone (FCCP) at concentrations up to 0.5 µM. In the presence of 0.5 mM cyanide, no residual respiration was observed.

The maximal activity of cytochrome *c* oxidase (COX, complex IV) in cells was determined by measuring the OCR with up to 2 mM N,N,N′,N′-tetramethyl-*p*-phenylenediamine (TMPD) in the presence of 10 µM antimycin A and 8 mM ascorbate, as described previously [23]. The assay was performed with a final cell protein concentration of 0.1 mg/mL.

To evaluate mitochondrial respiratory function, cells were permeabilized with 0.02% digitonin and incubated with a mixture of respiratory substrates. Complex I-linked respiration was determined using 5 mM malate and 5 mM pyruvate. Complex II-linked respiration was assessed using 5 mM succinate in the presence of 2 μM rotenone. Assays were conducted at 37 °C in 0.6 mL of incubation medium containing 150 mM sucrose, 2 mM MgCl_2_, 2.5 mM KH_2_PO_4_, 20 mM Tris-HCl buffer (pH 7.2), 0.1% bovine serum albumin (BSA), and a final cell protein concentration of 3 mg/mL. Phosphorylating respiration (state 3) was measured following the addition of 150 µM ADP. Non-phosphorylating respiration (state 4) was measured in the presence of oligomycin (1 µg/mg protein), an inhibitor of ATP synthase. The respiratory control ratio in the presence of oligomycin (RCRoligo) was calculated as the ratio of the state 3 to state 4 respiration rates.

### 2.10. Statistical Analysis

Data are presented as the mean ± standard deviation (SD) of 4–8 independent experiments using cell suspensions or cytoplasmic fractions. Each parameter was measured in at least two replicates. Normal distribution of data was evaluated using Q–Q plots and the Shapiro–Wilk test. Statistical analyses were performed using one-way analysis of variance (ANOVA) with Tukey’s post hoc test. Differences were considered statistically significant at *p* < 0.05, with significance levels indicated as follows: *p* < 0.05 (*), *p* < 0.01 (**), and *p* < 0.001 (***). Statistical values for all analyses, particularly *F* values of ANOVA and exact *p* values from post hoc comparisons, are provided in the Supplementary Data S1–S7.

## 3. Results

Lipophilic statins can cross the blood–brain barrier more effectively than hydrophilic statins, allowing them to affect cells of the CNS, such as astrocytes [24,25,26,27]. Therefore, we selected two commonly prescribed lipophilic statins for this study: atorvastatin, administered as the active hydroxyacid, and simvastatin, a lactone prodrug that undergoes first-pass hepatic hydrolysis to the pharmacologically active β-hydroxyacid metabolite [28]. Before in vitro use, simvastatin must be chemically activated to the active acid form (Section 2.1). We presented preliminary data on the effects of statins on total CoQ levels, cellular respiration, ROS formation, and ATP levels in the form of a conference abstract [29].

### 3.1. Concentration-Dependent Effects of Statins on Astrocytes’ Viability and CoQ9 and CoQ10 Levels

The nanomolar concentrations of statins used in our experiments were chosen to reflect clinically relevant plasma concentrations measured in patients receiving therapeutic doses of statins [30,31,32,33]. Our initial goal was to determine the concentrations of atorvastatin and simvastatin that significantly alter CoQ9 (the predominant CoQ form in rodents) and CoQ10 levels while maintaining astrocyte viability. Thus, we exposed astrocytes to increasing concentrations of these statins up to 250 nM (Figure 1). Neither atorvastatin nor simvastatin affected cell viability at concentrations up to 200 nM (Figure 1a). However, treatment at 250 nM significantly decreased the number of viable astrocytes. We observed a gradual, concentration-dependent reduction in CoQ9 and CoQ10 with increasing statin doses (Figure 1b). At the highest concentration tested (250 nM), CoQ10, the less abundant isoform that constitutes ~18% of total cellular CoQ (CoQ9 + CoQ10), became undetectable. Therefore, we performed all subsequent experiments using astrocytes cultured for 6 days under control conditions or in the presence of 200 nM atorvastatin or simvastatin, as these concentrations preserved cell viability while significantly decreasing intracellular CoQ9 (by ~30%) and CoQ10 (by ~70%) levels; total cellular CoQ content decreased by ~35% (Figure 1c).

The statin-induced decrease in cellular CoQ9 and CoQ10 levels was accompanied by a substantially larger decrease in the reduced CoQ pools of CoQ9 (by ~60%) and CoQ10 (by ~75%) than in the oxidized pools of CoQ9 (by ~20%) and CoQ10 (by ~65%, Figure 1c). In untreated astrocytes, the reduced CoQ (CoQH_2_) forms, which have antioxidant properties, accounted for ~25% of the total (oxidized + reduced) CoQ9 and CoQ10 pools. The effects of 200 nM atorvastatin and simvastatin were similar.

Our results indicate that prolonged exposure of astrocytes to elevated statin concentrations (>200 nM) impairs their viability, suggesting a potential cytotoxic effect. Moreover, they provide the first evidence that statin treatment significantly decreases CoQ9 and CoQ10 levels in astrocytes, especially their reduced forms, suggesting that disruption of CoQ homeostasis in astrocytes may contribute to reduced viability. Because CoQ is a potent lipid-soluble antioxidant that protects cell membranes from oxidative damage, we next investigated whether statin-induced CoQ depletion could induce oxidative stress in astrocytes.

### 3.2. Statin-Induced CoQ Depletion in Astrocytes Upregulates Cytoprotective Stress-Response Proteins and Reduces ROS Levels

**Figure 2:**
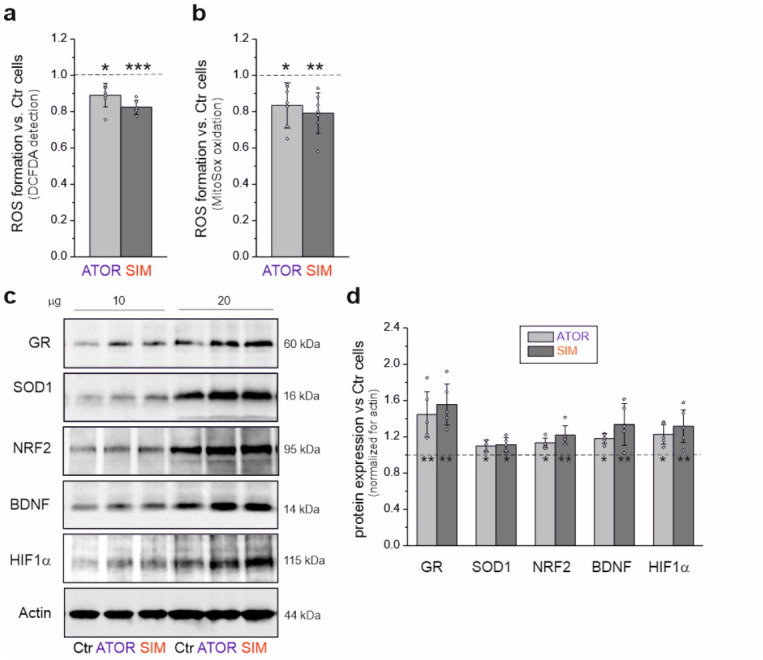
Effects of 200 nM atorvastatin (ATOR) and simvastatin (SIM) on (**a**) total and (**b**) mitochondrial ROS production and (**c**,**d**) antioxidant defense or redox-regulated factors in statin-treated astrocytes. (**c**) Representative Western blots (cropped blots); the µg values given refer to the amount of protein loaded into each lane. (**d**) Analysis of protein levels. Data from the 10 µg and 20 µg protein loading conditions were obtained from the same experimental sample. Therefore, results from both loading conditions were combined and treated as a single experimental sample for statistical analysis. (**a**,**b**) The data shown are the mean ± SD across 6–7 replicates, and comparisons were performed using one-way ANOVA. (**d**) The data shown are the mean ± SD across five replicates, compared using one-way ANOVA. Significance: *, *p* < 0.05; **, *p* < 0.01; ***, *p* < 0.001 vs. control astrocytes (Ctr) (dashed lines). Abbreviations: BDNF, brain-derived neurotrophic factor; GR, glutathione reductase; HIF1α, hypoxia-inducible factor 1α; NRF2, nuclear factor erythroid 2-related factor 2/NFE2-like bZIP transcription factor 2; SOD1, superoxide dismutase 1.

To assess the effect of statin treatment on astrocyte redox homeostasis, we quantified total and mitochondrial ROS levels in astrocytes after exposure to 200 nM atorvastatin or simvastatin. Surprisingly, the reduction in CoQ levels (Figure 1b,c) was accompanied by decreased ROS production. Both total cellular and mitochondrial ROS levels were ~11–20% lower in treated astrocytes compared to untreated astrocytes (Figure 2a,b).

Attenuation of oxidative stress was associated with an increased expression of key antioxidant enzymes in statin-treated astrocytes. GR levels increased by ~50% and SOD1 levels by ~12% compared to the control astrocytes (Figure 2c,d). Similarly, proteins involved in cellular stress adaptation and survival pathways were upregulated. Levels of HIF1α, a transcription factor that mediates cellular adaptation to hypoxia and coordinates metabolic and survival responses, increased by ~23–32% in statin-treated astrocytes (Figure 2c,d). Similarly, levels of BDNF, a neurotrophin essential for neuronal survival, differentiation, and synaptic plasticity, increased by ~18–34% in statin-treated astrocytes (Figure 2c,d). Moreover, levels of NRF2, the key regulator of antioxidant and cytoprotective gene expression, increased by 13–22% in statin-treated astrocytes (Figure 2c,d). The effects of atorvastatin and simvastatin were statistically comparable for all measured parameters.

These results indicate that despite a significant reduction in cellular CoQ content, statin-treated astrocytes activate a cytoprotective program characterized by increased expression of antioxidant and stress-responsive proteins, including HIF1α, NRF2, and BDNF, leading to reduced ROS production.

### 3.3. Statin Treatment Enhances the Aerobic and Anaerobic Respiratory Capacities of Astrocytes

**Figure 3:**
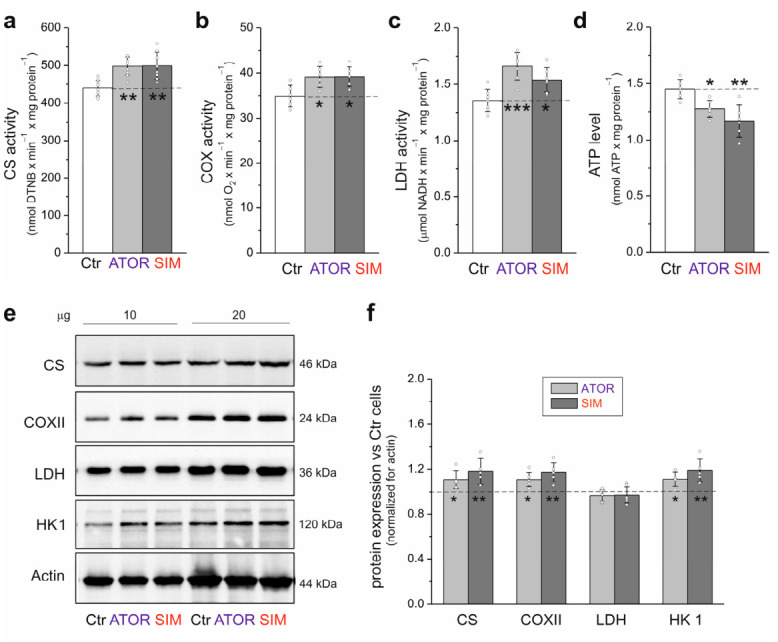
Effect of 6-day astrocyte culture with 200 nM atorvastatin (ATOR) and simvastatin (SIM) on markers of mitochondrial and anaerobic respiration. Activities of (**a**,**b**) aerobic and (**c**) anaerobic respiration markers and (**d**) ATP levels. (**e**) Representative Western blots (cropped blots) and (**f**) analysis of protein expression. Data from the 10 µg and 20 µg protein loading conditions were obtained from the same experimental sample. Therefore, results from both loading conditions were combined and treated as a single experimental sample for statistical analysis. (**a**–**d**) The data shown are the mean ± SD across 6–7 replicates, and the comparisons were performed using one-way ANOVA. (**f**) The data shown are the mean ± SD across 5–6 replicates, compared using one-way ANOVA. Significance: *, *p* < 0.05; **, *p* < 0.01; ***, *p* < 0.001 vs. control astrocytes (Ctr) (dashed lines). Abbreviations: COX, cytochrome *c* oxidase; COXII, COX subunit II; CS, citric synthase; HK1, hexokinase 1; LDH, lactate dehydrogenase.

CS, the initial enzyme of the tricarboxylic acid (TCA) cycle, and COX, complex IV of the respiratory chain, are markers of mitochondrial oxidative function. Relative to untreated astrocytes, statin-exposed astrocytes showed modest increases (~11–17%) in the activity (Figure 3a,b) and protein levels (Figure 3e,f) of both CS and COX. These results indicate that statins increase the oxidative capacity of both the TCA cycle and the mitochondrial respiratory chain. Furthermore, the level of HK1, a key glycolytic enzyme, and the activity (but not the level) of LDH, which catalyzes the conversion of pyruvate to lactate, were elevated in statin-treated astrocytes (Figure 3c,e,f).

Therefore, our findings suggest that statins increase mitochondrial oxidative capacity and anaerobic respiration in astrocytes. However, despite this increase in respiratory activity, statin-treated astrocytes showed a ~14–27% decrease in ATP levels compared to untreated astrocytes (Figure 3d).

### 3.4. Statins Enhance Mitochondrial Fission and Influence Mitochondrial Turnover in Astrocytes

**Figure 4:**
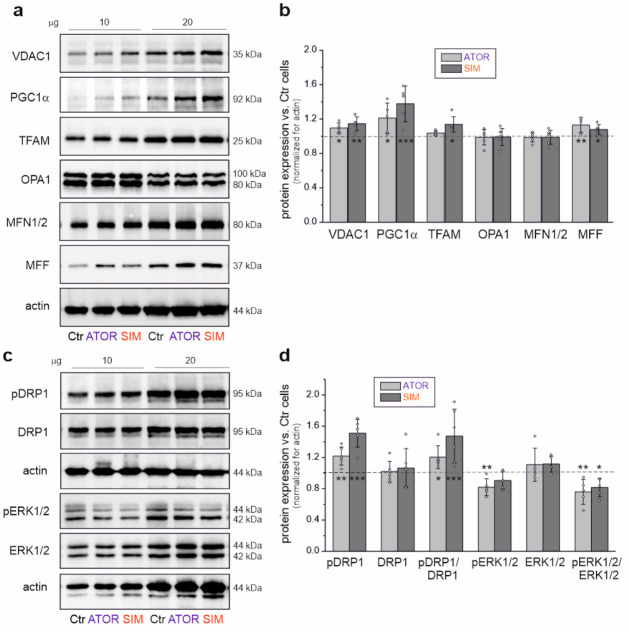
Markers of mitochondrial dynamics in astrocytes cultured with 200 nM atorvastatin (ATOR) and simvastatin (SIM). (**a**,**c**) Representative Western blots (cropped) and (**b**,**d**) analyses of protein levels. Data from the 10 µg and 20 µg protein loading conditions were obtained from the same experimental sample. Therefore, results from both loading conditions were combined and treated as a single experimental sample for statistical analysis. The data shown are the mean ± SD across 5–6 replicates, compared using one-way ANOVA. Significance: *, *p* < 0.05; **, *p* < 0.01; ***, *p* < 0.001 vs. control astrocytes (Ctr) (dashed lines). Abbreviations: (**a**,**b**) MFN1/2, mitofusin 1/2; MFF, mitochondrial fission factor; OPA1, OPA1 mitochondrial dynamin-like GTPase; PGC1α, peroxisome proliferator-activated receptor γ coactivator 1α; TFAM, transcription factor A, mitochondrial; VDAC1, voltage-dependent anion-selective channel 1; (**c**,**d**) DRP1, total dynamin related protein 1; pDRP1, phosphorylated DRP1; ERK1/2, total extracellular signal-regulated protein kinases 1/2; p-ERK1/2, phosphorylated ERK1/2.

Statin treatment increased the activity and protein levels of the mitochondrial markers CS and COX by up to ~17% (Figure 3), suggesting increased mitochondrial abundance in astrocytes. This observation was further supported by a ~10–15% increase in the expression of VDAC1, a protein located in the outer mitochondrial membrane and commonly used as an indicator of mitochondrial abundance (Figure 4a,b).

To further characterize the effects of statins on mitochondrial dynamics, we analyzed proteins involved in mitochondrial biogenesis, fission, and fusion. Statin treatment increased the expression of key regulators of mitochondrial biogenesis. Levels of PGC1α increased by ~22–38% in atorvastatin- and simvastatin-treated astrocytes, whereas levels of TFAM, which plays a central role in mitochondrial DNA (mtDNA) replication and transcription, increased by ~13% only in simvastatin-treated astrocytes (Figure 4a,b). A slight increase in mtDNA copy number [34,35] was also observed (Supplementary Figure S5), confirming enhanced mitochondrial biogenesis in statin-treated astrocytes.

Moreover, the levels of proteins that serve as markers of mitochondrial fission were increased, including MFF (~8–14%, Figure 4a,b) and active phospho-DRP1 (~20–50%, Figure 4c,d), indicating increased mitochondrial fission. In contrast, the levels of mitochondrial fusion markers, including OPA1 and MFN1/2, remained unchanged (Figure 4a,b).

Because ERK1/2 can be involved in both astrocyte survival [36] and DRP1-dependent mitochondrial fission [37], we examined their activation status after statin treatment. The levels of active phospho-ERK1/2 were reduced, although the difference was significant only in atorvastatin-treated astrocytes (Figure 4c,d). However, the ratio of phospho-ERK1/2 to total ERK1/2 was markedly decreased (~20–25%) in astrocytes treated with both atorvastatin and simvastatin compared to the control astrocytes, suggesting that statins downregulate the ERK1/2 signaling pathway.

These results indicate a statin-induced shift in mitochondrial turnover characterized by enhanced biogenesis and fission activity, suggesting an ERK1/2-independent pathway for mitochondrial remodeling.

### 3.5. Statins Alter Astrocyte Energy Metabolism by Reducing Mitochondrial Coupling Efficiency and ATP Production

**Figure 5:**
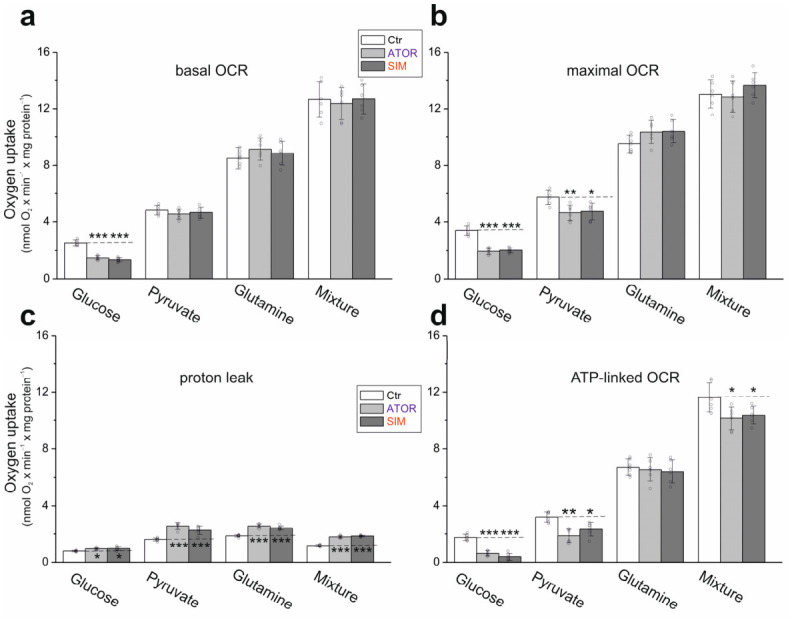
Effect of 200 nM atorvastatin (ATOR) and simvastatin (SIM) on mitochondrial oxidative metabolism in astrocytes. (**a**) Basal OCR, (**b**) maximal OCR, (**c**) proton leak, and (**d**) ATP-linked OCR using 5.5 mM glucose, 5 mM pyruvate, 4 mM glutamine, or a mixture thereof. The data shown are the mean ± SD across six replicates, compared using one-way ANOVA. Significance: *, *p* < 0.05; **, *p* < 0.01; ***, *p* < 0.001 vs. control astrocytes (Ctr) (dashed lines).

To investigate changes in the aerobic metabolism activity, we measured the oxygen consumption rate (OCR) of astrocytes exposed to 200 nM atorvastatin or 200 nM simvastatin using various reducing substrates: glucose, pyruvate, glutamate, and their mixture (Figure 5). During the oxidation of glutamate (amino acid–derived substrate) and during the oxidation of a mixture of all tested substrates, statin-treated astrocytes did not show any significant changes in basal or maximal OCR compared to control cells (Figure 5a,b). However, when cells were supplied with weaker carbohydrate substrates such as glucose or pyruvate, the maximal OCR was reduced, and the basal OCR was also lower when glucose was oxidized. This reduction in carbohydrate substrate oxidation indicates a metabolic reprogramming consistent with a shift towards glycolytic metabolism, as also indicated by increased HK1 levels and LDH activity (Figure 3).

With all substrates tested, statin-treated astrocytes showed increased non-ATP-linked respiration (proton leak) and decreased ATP-linked OCR (except for glutamate; Figure 5c,d), suggesting reduced mitochondrial coupling efficiency and reduced ATP production.

**Figure 6:**
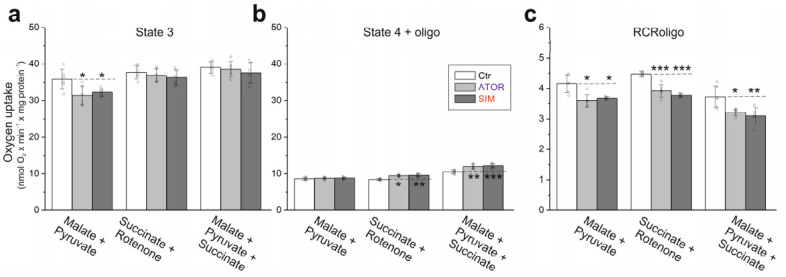
Respiratory rates (**a**,**b**) and respiratory control ratios (**c**) in permeabilized control (Ctr), atorvastatin-treated (ATOR), and simvastatin-treated (SIM) astrocytes using malate and pyruvate, succinate (plus rotenone), or a mixture of malate, pyruvate, and succinate. The data shown are the mean ± SD across six replicates, compared using one-way ANOVA. Significance: *, *p* < 0.05; **, *p* < 0.01; ***, *p* < 0.001 vs. control astrocytes (Ctr) (dashed lines). Abbreviations: State 3, phosphorylating respiration in the presence of ADP; State 4 + oligo, non-phosphorylating respiration in the presence of oligomycin; RCRoligo, State 3 vs. State 4 in the presence of oligomycin.

Measuring the OCR in permeabilized cells allows assessment of mitochondrial respiration driven by complex I substrates (malate and pyruvate) and complex II substrates (succinate). Permeabilized astrocytes exposed to statins showed an ~15% reduction in ADP-stimulated (phosphorylating) respiration during malate and pyruvate oxidation. In contrast, succinate oxidation and respiration driven by combined complex I and II substrates remained unchanged (Figure 6a). Since statin-treated astrocytes showed increased mitochondrial oxidative capacity and biogenesis (Figure 3 and Figure 4), unchanged or even reduced phosphorylating respiration indicates impaired mitochondrial bioenergetic functions. Interestingly, when permeabilized statin-treated astrocytes oxidized succinate alone or a mixture of complex I and II substrates, oligomycin-insensitive (non-phosphorylating) respiration increased by 12–15% (Figure 6b).

In addition, the respiratory control ratio measured in the presence of oligomycin (RCRoligo) was significantly decreased (Figure 6c). RCRoligo reflects the maximal factorial increase in mitochondrial OCR, attributed to ADP phosphorylation above proton leak-dependent OCR when ATP synthase is inhibited by oligomycin. These results indicate that statin-treated astrocytes exhibit increased mitochondrial respiration unrelated to ATP synthesis, similar to that measured with intact astrocytes (Figure 5c).

Altogether, these results indicate that statins change energy metabolism in astrocytes. Statin-treated astrocytes exhibit increased mitochondrial uncoupling (proton leak), which reduces the efficiency of oxidative phosphorylation and thereby reduces ATP production. They also suggest potential inhibition of the respiratory chain, particularly during the oxidation of complex I substrates.

### 3.6. CoQ10 Supplementation Increases CoQ Levels and Restores ATP Levels but Does Not Alter ROS Production in Statin-Treated Astrocytes

**Figure 7:**
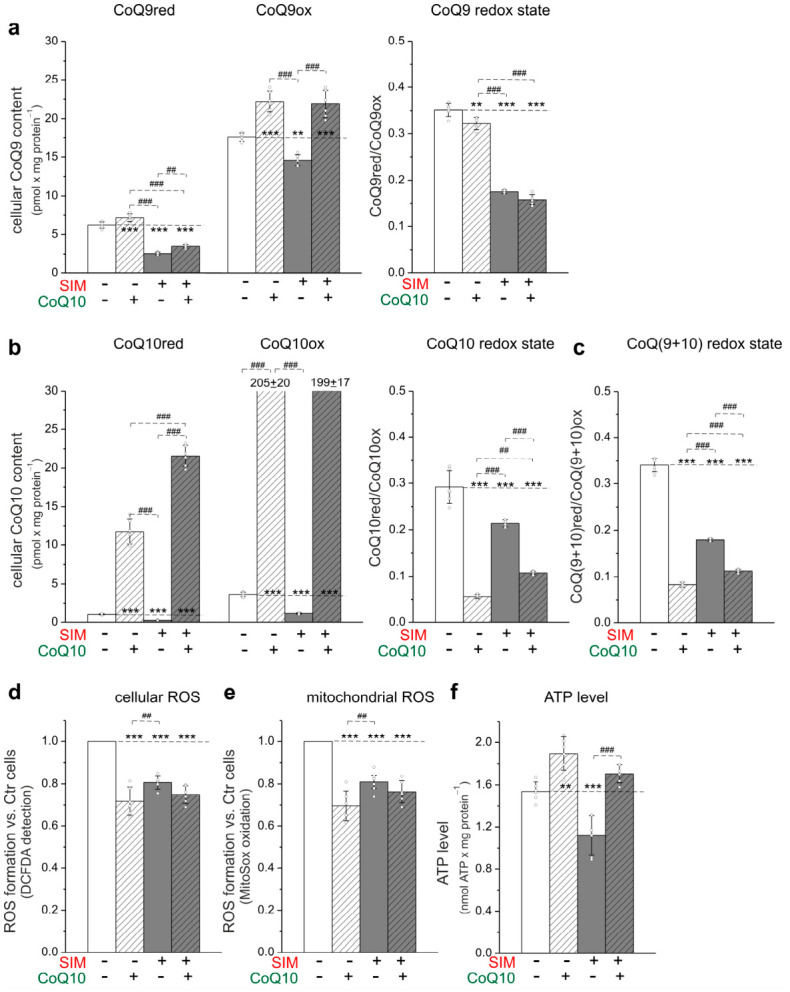
Effects of 3.4 µM CoQ10 supplementation on cellular (**a**) CoQ9 and (**b**) CoQ10 content and redox state, (**c**) total CoQ (CoQ9 + CoQ10) redox state, (**d**) total and (**e**) mitochondrial ROS production, and (**f**) ATP levels in simvastatin (SIM)-treated astrocytes. Astrocytes were cultured for 6 days with or without 200 nm SIM and/or 3.4 µM CoQ10. (**a**,**b**) Reduced (red), oxidized (ox) forms of CoQ9 and CoQ10; (**a**–**c**), redox state, CoQred/CoQox. (**a**–**f**) The data shown are the mean ± SD across six replicates, compared using one-way ANOVA. Significance: **, *p* < 0.01; ***, *p* < 0.001 vs. control astrocytes (Ctr) (dashed lines). Hash symbols indicate pairwise comparisons between the groups connected by dashed brackets (^##^, *p* < 0.01; ^###^, *p* < 0.001).

Next, we examined the effect of CoQ10 supplementation on the CoQ redox state in statin-treated astrocytes (Figure 7a,b). Because the effects of atorvastatin and simvastatin on astrocytes did not differ statistically in the earlier experiments (Figure 1, Figure 2, Figure 3, Figure 4, Figure 5 and Figure 6), we selected simvastatin for the CoQ supplementation assays. We cultured astrocytes in the presence or absence of 3.4 µM CoQ10. This concentration was chosen because it corresponds to the plasma concentration observed in individuals taking CoQ10 supplementation [20], thus providing a physiologically relevant level of exposure.

In untreated astrocytes, CoQ10 supplementation increased both the reduced and oxidized forms of CoQ9 (by ~16% and ~26%, respectively) and CoQ10 (by ~12-fold and ~50%, respectively; Figure 7a,b). These increases resulted in a decrease in the redox state (CoQ_red_/CoQ_ox_) of both CoQ species, particularly CoQ10. As described above, simvastatin alone decreased the levels of reduced and oxidized CoQ9 (by ~60% and ~20%, respectively) and reduced and oxidized CoQ10 (by ~75% and ~65%, respectively), leading to a decrease in the redox state of both CoQs (Figure 7a,b). In simvastatin-treated astrocytes, CoQ10 supplementation elevated the reduced and oxidized forms of both CoQ9 (by ~40% and ~50%, respectively) and CoQ10 (by ~85-fold and ~170-fold, respectively). Altogether, these changes led to a further decrease in the redox state of CoQ10 (Figure 7b) and the redox state of the total CoQ pool (CoQ9 + CoQ10, Figure 1c) in astrocytes treated with simvastatin and CoQ10 compared to astrocytes treated with simvastatin alone.

Treatment with simvastatin alone, supplementation with CoQ10 alone, and combined treatment with simvastatin and CoQ10 resulted in a decrease in cellular and mitochondrial ROS levels in astrocytes compared to untreated astrocytes (Figure 7d,e). CoQ10 supplementation did not result in a further significant decrease in ROS levels when coadministered with simvastatin. Thus, although CoQ10 supplementation decreases the redox state of CoQ10 and the total pool of CoQ9 and CoQ10 in simvastatin-treated astrocytes, this further change in redox state does not translate into a further decrease in ROS production.

Interestingly, CoQ10 supplementation increased ATP levels in both untreated and simvastatin-treated astrocytes (Figure 7f). Thus, CoQ10 supplementation restored the ATP levels that were lowered by statin treatment.

Overall, these results demonstrate that CoQ10 supplementation in astrocytes clearly (i) alters cellular CoQ profiles by lowering the CoQ redox state, (ii) reduces ROS levels independently of statin treatment, and (iii) restores ATP content, counteracting statin-induced disturbances in energy metabolism.

## 4. Discussion

The effects of statins on astrocyte oxidative metabolism remain poorly understood. In this study, a 6-day treatment of rat CTX TNA2 astrocytes with 200 nM atorvastatin or simvastatin resulted in comparable metabolic changes, without many significant differences between them.

Several studies have linked statin-induced cytotoxicity to inhibition of the mevalonate pathway and subsequent depletion of essential metabolites, including isoprenoid intermediates and CoQ [1,2,3,7,10]. Exposure of astrocytes to higher concentrations of atorvastatin and simvastatin (>200 nM) decreased cell viability, suggesting potential concentration-dependent cellular toxicity (Figure 1). This observation is consistent with previous studies showing that statins can exert cytotoxic effects on astrocytes when used at supraphysiological concentrations (>1 µM), indicating that isoprenoids (mainly geranylgeranyl pyrophosphate), products of the mevalonate pathway, play a key role in astrocyte survival [14].

Statins are known to lower CoQ levels, reflecting inhibition of the mevalonate pathway, which is necessary for CoQ biosynthesis [7,8,38]. Our study provides the first direct evidence that statin treatment significantly disrupts CoQ homeostasis in astrocytes, resulting in a marked decrease in CoQ9 and CoQ10 levels (total cellular CoQ content decreased by ~35%, Figure 1b), with a particularly pronounced decrease in their reduced forms (by ~60–75%, Figure 1c). Although CoQ deficiency has been well documented, especially in skeletal muscle in the context of statin-induced myopathy, its effect on astrocytes has not yet been directly demonstrated [39]. The preferential loss of reduced CoQ (CoQH_2_) observed in our study is particularly relevant, as it acts as a potent lipid-soluble antioxidant that prevents lipid peroxidation and protects cell membranes from oxidative damage [9]. However, despite a significant reduction in total cellular CoQ content, accompanied by the elimination of the reduced CoQH_2_ pool (Figure 1), statin-treated astrocytes showed reduced intracellular and mitochondrial ROS levels and not increased oxidative stress (Figure 2a,b). Thus, our findings demonstrate that astrocytes generated a potent cytoprotective response that limits ROS accumulation. Previous studies indicate that statin effects are tissue-specific. Unlike in skeletal muscle, statins reduce ROS production and increase antioxidant enzyme expression in cardiac muscle [40] and in astrocytes (our study), thereby reducing oxidative stress.

Specifically, in astrocytes, statins activated compensatory antioxidant pathways, including NRF2 signaling and upregulation of antioxidant enzymes (GR and SOD1, Figure 2). Activation of the transcription factor NRF2 is a well-documented adaptive response to mitochondrial and redox stress that stimulates the expression of antioxidant and detoxifying enzymes, also in astrocytes [41,42]. Statins are known to exert their antioxidant effects by modulating NRF2 signaling [43]. In addition to the aforementioned antioxidant defense mechanisms, statin-induced CoQ deficiency in astrocytes was associated with increased expression of BDNF and HIF1α (Figure 2), which are factors involved in cellular adaptation to metabolic stress. Our observation of increased BDNF levels in astrocytes (Figure 2) is consistent with previous studies demonstrating mevalonate-independent upregulation of neurotrophin expression, which supports astrocyte and neuronal survival and neuroprotective signaling [44].

In our study, statin treatment increased HIF1α and HK1 levels and LDH activity (Figure 2 and Figure 3), indicating the activation of a functional glycolytic response in astrocytes. A reduction in the oxidation of carbohydrate metabolism substrates, such as glucose and pyruvate, was also observed in statin-treated astrocytes (Figure 5b), confirming metabolic reprogramming consistent with a shift towards glycolytic metabolism. Previous studies have shown that HIF1α activation in astrocytes promotes a shift towards glycolytic metabolism by upregulating key glycolytic enzymes, including HK1, and decreasing reliance on mitochondrial oxidative phosphorylation [45,46,47,48]. Moreover, HIF1α activation can inhibit mitochondrial respiration and promote mechanisms (e.g., mitophagy) that limit mitochondrial ROS production, thus contributing to metabolic adaptation under stress conditions [49,50]. Consistent with these observations, our findings indicate that in astrocytes, statin-induced HIF1α activation was associated with decreased mitochondrial ROS production (Figure 2), increased mitochondrial fission (Figure 5), and decreased mitochondrial respiration with complex I substrates (Figure 6).

Statin-treated astrocytes exhibited increased levels of mitochondrial fission markers (MFF and phospho-DRP1), whereas the expression of mitochondrial fusion proteins (OPA1 and MFN1/2) remained unchanged (Figure 4). The balance between mitochondrial fission and fusion enables the mitochondrial network to adapt to metabolic demands and facilitates the redistribution of mitochondrial contents [37]. In our experiments, the increase in fission-related markers may reflect enhanced mitophagy-mediated mitochondrial clearance. This effect appears to occur independently of downregulated ERK1/2 signaling (Figure 4), a pathway known to regulate mitochondrial dynamics and apoptosis [37]. Because statins inhibit HMG-CoA reductase and block the prenylation and activation of small GTPases, they may reduce prenylation-dependent ERK1/2 signaling and contribute to metabolic reprogramming. Overall, statin-induced changes in mitochondrial turnover in astrocytes may promote the removal of damaged mitochondria via mitophagy and help preserve mitochondrial function. However, further studies are needed to confirm statin-induced changes in astrocyte mitochondrial turnover, such as by directly assessing mitochondrial morphology. Several studies have shown that inhibiting the mevalonate pathway alters mitochondrial dynamics and turnover across various cell types, in part by altering the activity of small GTPases and signaling pathways regulating fission, fusion, and autophagy [7]. Statins have been shown to induce mitochondrial fragmentation, stimulate mitophagy, and reduce the accumulation of dysfunctional mitochondria, which may help maintain mitochondrial efficiency and limit oxidative stress under conditions of metabolic stress.

Despite the increased expression of mitochondrial fission markers (MFF and phospho-DRP1), the increased expression of mitochondrial biogenesis markers, including mitochondrial proteins (CS, COX, and VDAC1) and transcription factors (PGC1α and TFAM) (Figure 3 and Figure 4), indicates a statin-induced change in mitochondrial turnover, accompanied by an increase in mitochondrial biogenesis. However, despite the greater total respiratory capacity, as evidenced by COX activity (Figure 3b), no increase in the maximal oxidative respiration activity was observed in intact statin-treated astrocytes (Figure 5b), indicating an impairment of the mitochondrial respiratory chain. Therefore, statin-induced changes in mitochondrial turnover cannot fully compensate for disturbances in the mitochondrial respiratory chain in astrocytes. Moreover, significantly reduced phosphorylating respiration during oxidation of complex I substrates (malate and pyruvate) was observed in permeabilized astrocytes treated with statins (Figure 6a). Furthermore, statin-treated astrocytes showed increased mitochondrial uncoupling (proton leak) (Figure 5d and Figure 6b,c), decreased ATP-coupled respiration (Figure 5d and Figure 6a), and decreased ATP levels (Figure 3d), indicating impaired energy metabolism with reduced mitochondrial oxidative phosphorylation efficiency and decreased ATP production. Altogether, these results indicate for the first time that statins alter oxidative metabolism in astrocytes, likely due to CoQ deficiency. Our results are consistent with studies showing that statins can adversely affect mitochondrial respiratory chain activity and oxidative phosphorylation in various cell types, often in combination with reduced CoQ synthesis due to inhibition of HMG-CoA reductase [7,8].

Our experiments indicate that astrocytes are characterized by significant metabolic plasticity, and in response to statin treatment, reducing ROS production and oxidative stress (Figure 2a,b) is more important than maintaining ATP levels (Figure 3d). This promotion of redox balance is consistent with the well-known role of astrocytes as key regulators of brain redox homeostasis and their greater reliance on glycolysis compared to neurons, which allows them to tolerate lower mitochondrial ATP production [11,51]. Statins have been shown to reduce ROS production and enhance antioxidant defenses in many cell types, including vascular endothelial, cardiac, and renal cells, suggesting protective adaptation rather than solely mitochondrial damage [7,43,52,53,54,55,56]. Thus, our findings indicate that reducing oxidative stress may be a major adaptive priority for astrocytes exposed to statins.

We reported for the first time the effects of CoQ10 supplementation on CoQ content and redox state (CoQred/CoQox), ROS production, and ATP levels in statin-treated astrocytes (Figure 7). Simvastatin alone decreased the levels of reduced and oxidized CoQ9 and CoQ10, resulting in a lower redox state for both CoQ forms. In simvastatin-treated astrocytes, CoQ10 supplementation increased levels of reduced and oxidized forms of CoQ, particularly CoQ10, compared to statin treatment alone. These increases were accompanied by a further decrease in the redox state of CoQ10 and the total CoQ pool (CoQ9 + CoQ10), suggesting that the additional CoQ10 remains more oxidized. Nevertheless, the increase in the pool of reduced CoQ forms indicates an overall increase in antioxidant potential.

Treatment with simvastatin, exogenous CoQ10, and their combination reduced intracellular and mitochondrial ROS levels in astrocytes compared to untreated astrocytes (Figure 7d,e). Notably, CoQ10 supplementation did not further reduce ROS production during simvastatin treatment, suggesting that ROS production may already be minimized by statin treatment or that further changes in CoQ redox status do not translate into additional ROS suppression, thus highlighting the antioxidant effects of statins on astrocytes. However, exogenous CoQ10 has previously been shown to protect astrocytes from ultraviolet B-induced ROS production and block mitochondrial-dependent cell death pathways, highlighting the importance of intact CoQ-dependent antioxidant capacity for astrocyte survival [57]. In our study, CoQ10 supplementation significantly increased ATP levels in both untreated and simvastatin-treated astrocytes, restoring ATP production suppressed by statin treatment (Figure 7f). This observation supports the key role of CoQ10 in mitochondrial electron transport and energy production [58], indicating that its main effect in astrocytes is bioenergetic rather than antioxidant. Therefore, CoQ10 supplementation in astrocytes may improve bioenergetic function under conditions of limited CoQ10 availability due to statins. Altogether, these results indicate that the primary benefit of CoQ10 supplementation in astrocytes is support for mitochondrial energy metabolism rather than further inhibition of ROS production.

Given the widespread chronic use of statins in elderly patients, including those with neurodegenerative diseases, our findings may have important implications. Neurodegenerative diseases are often associated with mitochondrial dysfunction, oxidative stress, and chronic inflammatory activation [59,60]. Reactive astrocytes play a key role in these processes and may contribute to neuronal dysfunction and neurodegeneration [61]. In addition to their lipid-lowering effects, statins exhibit pleiotropic anti-inflammatory and immunomodulatory effects, including inhibition of proinflammatory cytokines, suppression of microglial activation, and protection of the blood–brain barrier integrity, which may contribute to reduced neuroinflammation [62]. Our findings indicate that prolonged exposure to statins causes CoQ deficiency, mitochondrial remodeling, and reduced ATP production in astrocytes, but also activation of adaptive antioxidant pathways. Although these adaptations may support redox homeostasis, the associated bioenergetic disturbances may impact astrocyte function in neurodegenerative diseases. Therefore, CoQ10 supplementation may be particularly beneficial in neurodegenerative diseases [63], especially during long-term statin treatment, helping to maintain mitochondrial energy metabolism.

## 5. Conclusions

**Figure 8:**
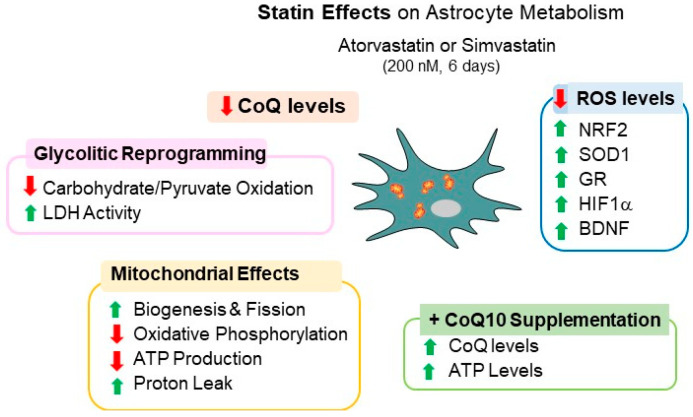
Schematic representation of the cellular and molecular changes associated with metabolic reprogramming induced by CoQ deficiency in astrocytes.

This study demonstrated, for the first time, that prolonged 6-day exposure of astrocytes to clinically relevant concentrations of statins significantly alters astrocyte energy metabolism by disrupting CoQ homeostasis and oxidative respiratory function and by altering mitochondrial turnover (Figure 8). Statin-induced inhibition of the mevalonate pathway led to a deficit of CoQ, a key antioxidant and mitochondrial electron carrier, but astrocytes did not develop oxidative stress. Instead, they triggered a coordinated cytoprotective program involving NRF2 signaling, induction of antioxidant enzymes, metabolic reprogramming toward glycolysis, and alteration of mitochondrial turnover, thereby prioritizing preservation of redox balance over ATP production. These results indicate that the depletion of CoQ levels in astrocytes may be the main mechanism driving metabolic adaptation to statins and highlight the high metabolic plasticity of astrocytes. We further demonstrated that CoQ10 supplementation replenishes CoQ stores and restores ATP levels in statin-treated astrocytes, although it does not additionally inhibit ROS production, suggesting that its primary role is bioenergetic support rather than enhancing antioxidant defense. Given the essential role of astrocytes in neuronal metabolism and supporting redox reactions, statin-induced changes in astrocyte CoQ homeostasis and mitochondrial function may have broader implications for brain energy homeostasis during long-term statin therapy and support the use of CoQ10 supplementation.

## Data Availability

All data generated or analyzed during this study are included in this published article (and its Supplementary Material files). The data are available from the corresponding author upon request.

## Supplementary Materials

The following supporting information can be downloaded at: https://www.mdpi.com/article/10.3390/antiox15060725/s1, Supplementary Figures S1–S5, Supplementary Data S1–S7. Figure S1: Uncropped images used to prepare Figure 2; Figure S2: Original images used to prepare Figure 3; Figure S3: Original images used to prepare Figure 4a; Figure S4: Original images used to prepare Figure 4b; Figure S5: Effect of 200 nM atorvastatin and simvastatin on mtDNA copy number; Data S1: *F* and *p* values obtained from post hoc ANOVA comparisons for the data presented in Figure 1; Data S2: *F* and *p* values obtained from post hoc ANOVA comparisons for the data presented in Figure 2; Data S3: *F* and *p* values obtained from post hoc ANOVA comparisons for the data presented in Figure 3; Data S4: *F* and *p* values obtained from post hoc ANOVA comparisons for the data presented in Figure 4; Data S5: *F* and *p* values obtained from post hoc ANOVA comparisons for the data presented in Figure 5; Data S6: *F* and *p* values obtained from post hoc ANOVA comparisons for the data presented in Figure 6; Data S7: *F* and *p* values obtained from post hoc ANOVA comparisons for the data presented in Figure 7.

## Abbreviations

The following abbreviations are used in this manuscript:

BDNF	Brain-derived neurotrophic factor
CoQ	Coenzyme Q
COX	Cytochrome *c* oxidase
CS	Citrate synthase
DRP1	Dynamin-related protein 1
ERK1/2	Extracellular signal-regulated protein kinase
GR	Glutathione reductase
HIF1α	Hypoxia-inducible factor 1-alpha
HK1	Hexokinase I
HMG-CoA reductase	3-Hydroxy-3-methylglutaryl-coenzyme A reductase
LDH	Lactate dehydrogenase
MFN1/2	Mitofusin 1/2
NRF2	Nuclear factor erythroid 2-related factor/ NFE2-like bZIP transcription factor 2
OPA1	OPA1 mitochondrial dynamin-like GTPase
PGC1α	Peroxisome proliferator-activated receptor γ coactivator 1α
ROS	Reactive oxygen species
SOD1	Superoxide dismutase 1
TFAM	Transcription factor A, mitochondrial
VDAC1	Voltage-dependent anion-selective channel protein 1
