# Supplementary material for: Statin-Induced Coenzyme Q Deficiency Induces Metabolic Reprogramming in Astrocytes
Source: Antioxidants (Basel). 2026 Jun 7;15(6):725. doi: 10.3390/antiox15060725 (PMC13295249; doi:10.3390/antiox15060725)
Supplement: Supplementary file 1 [file antioxidants-15-00725-s001.zip › Supplementary Data.pdf]

Fig. 1a	250 nM		
ANOVA	F	*p*	
	5,406	,017*	
post-hoc	Ctr	ATOR	SIM
Ctr		0,028593	0,033866
ATOR	0,028593		0,995889
SIM	0,033866	0,995889	

Fig. 1b	150 nM CoQ9		
ANOVA	F	*p*	
	7,336	,006*	
post-hoc	Ctr	ATOR	SIM
Ctr		0,007566	0,023390
ATOR	0,007566		0,836307
SIM	0,023390	0,836307	

	150 nM CoQ10		
ANOVA	F	p	
	159,6	,000*	
post-hoc	Ctr	ATOR	SIM
Ctr		0.000178	0.000178
ATOR	0.000178		0.196717
SIM	0.000178	0.196717	

	150 nM CoQ9+CoQ10		
ANOVA	F	*p*	
	24,17	,000*	
post-hoc	Ctr	ATOR	SIM
Ctr		0,000198	0,000322
ATOR	0,000198		0,623174
SIM	0,000322	0,623174	

	200 nM CoQ9		
ANOVA	F	*p*	
	21,36	,000*	
post-hoc	Ctr	ATOR	SIM
Ctr		0.000251	0.000327
ATOR	0.000251		0.936236
SIM	0.000327	0.936236	

	200 nM CoQ10		
ANOVA	F	p	
	257,6	,000*	
post-hoc	Ctr	ATOR	SIM
Ctr		0.000178	0.000178
ATOR	0.000178		0.417488
SIM	0.000178	0.417488	

	200 nM CoQ9+CoQ10		
ANOVA	F	p	
	68,59	,000*	
post-hoc	Ctr	ATOR	SIM
Ctr		0.000178	0.000178
ATOR	0.000178		0.806126
SIM	0.000178	0.806126	

	250 nM CoQ9		
ANOVA	F	p	
	46,17	,000*	
post-hoc	Ctr	ATOR	SIM
Ctr		0.000178	0.000178
ATOR	0.000178		0.828843
SIM	0.000178	0.828843	

	250 nM CoQ9+CoQ10		
ANOVA	F	p	
	172,2	,000*	
post-hoc	Ctr	ATOR	SIM
Ctr		0.000178	0.000178
ATOR	0.000178		0.708618
SIM	0.000178	0.708618	

Fig. 1c	CoQ9red		
ANOVA	F	*p*	
	380,2	,000*	
post-hoc	Ctr	ATOR	SIM
Ctr		0.000178	0.000178
ATOR	0.000178		0.769193
SIM	0.000178	0.769193	

	CoQ9tot		
ANOVA	F	*p*	
	40,57	,000*	
post-hoc	Ctr	ATOR	SIM
Ctr		0,000178	0,000180
ATOR	0,000178		0,880515
SIM	0,000180	0,880515	

	CoQ10red		
ANOVA	F	*p*	
	614,8	,000*	
post-hoc	Ctr	ATOR	SIM
Ctr		0.000178	0.000178
ATOR	0.000178		0.938130
SIM	0.000178	0.938130	

	CoQ10tot		
ANOVA	F	*p*	
	538,5	,000*	
post-hoc	Ctr	ATOR	SIM
Ctr		0,000178	0,000178
ATOR	0,000178		0,885904
SIM	0,000178	0,885904	

Data S1. *F* and *p* values obtained from post hoc ANOVA comparisons for the data presented in Figure 1.

Fig. 2a			
ANOVA	F	*p*	
	10,94	,001*	
post-hoc	Ctr	ATOR	SIM
Ctr		0.020123	0.000721
ATOR	0.020123		0.267627
SIM	0.000721	0.267627	

Fig. 2b			
ANOVA	F	*p*	
	8,296	,003*	
post-hoc	Ctr	ATOR	SIM
Ctr		0.015764	0.003443
ATOR	0.015764		0.758345
SIM	0.003443	0.758345	

Fig. 2d	GR		
ANOVA	F	*p*	
	13,38	,001*	
post-hoc	Ctr	ATOR	SIM
Ctr		0.007648	0.001020
ATOR	0.007648		0.452079
SIM	0.001020	0.452079	

	SOD1		
ANOVA	F	*p*	
	7,276	,009*	
post-hoc	Ctr	ATOR	SIM
Ctr		0.023736	0.011781
ATOR	0.023736		0.918898
SIM	0.011781	0.918898	

	NRF2		
ANOVA	F	*p*	
	11,54	,002*	
post-hoc	Ctr	ATOR	SIM
Ctr		0.038675	0.001352
ATOR	0.038675		0.163887
SIM	0.001352	0.163887	

	BDNF		
ANOVA	F	*p*	
	12,01	0,001*	
post-hoc	Ctr	ATOR	SIM
Ctr		0.045215	0.001148
ATOR	0.045215		0.118413
SIM	0.001148	0.118413	

	HIF-1α		
ANOVA	F	*p*	
	10,59	,002*	
post-hoc	Ctr	ATOR	SIM
Ctr		0.017805	0.002275
ATOR	0.017805		0.477859
SIM	0.002275	0.477859	

Data S2. *F* and *p* values obtained from post hoc ANOVA comparisons for the data presented in Figure 2.

**Fig. 3a**			
ANOVA	F	*p*	
	10,47	,001*	
post-hoc	Ctr	ATOR	SIM
Ctr		0.002686	0.001642
ATOR	0.002686		0.973022
SIM	0.001642	0.973022	

**Fig. 3b**			
ANOVA	F	*p*	
	6,820	,008*	
post-hoc	Ctr	ATOR	SIM
Ctr		0,016758	0,014646
ATOR	0,016758		0,997490
SIM	0,014646	0,997490	

**Fig. 3c**			
ANOVA	F	*p*	
	13,77	,000*	
post-hoc	Ctr	ATOR	SIM
Ctr		0,000293	0,015046
ATOR	0,000293		0,123654
SIM	0,015046	0,123654	

**Fig. 3d**			
ANOVA	F	*p*	
	10,87	,001*	
post-hoc	Ctr	ATOR	SIM
Ctr		0,030605	0,001040
ATOR	0,030605		0,214808
SIM	0,001040	0,214808	

Fig. 3f

	**CS**		
ANOVA	F	*p*	
	8,933	,004*	
post-hoc	Ctr	ATOR	SIM
Ctr		0.020594	0.004711
ATOR	0.020594		0.687608
SIM	0.004711	0.687608	

	**COXII**		
ANOVA	F	*p*	
	11,72	,002*	
post-hoc	Ctr	ATOR	SIM
Ctr		0.033749	0.001292
ATOR	0.033749		0.176158
SIM	0.001292	0.176158	

	**LDH**		
ANOVA	F	*p*	
	1.64	0,226	
post-hoc	Ctr	ATOR	SIM
Ctr		0.257630	0.329327
ATOR	0.257630		0.984365
SIM	0.329327	0.984365	

	**HK1**		
ANOVA	F	*p*	
	9,781	,003*	
post-hoc	Ctr	ATOR	SIM
Ctr		0.049085	0.002501
ATOR	0.049085		0.242517
SIM	0.002501	0.242517	

Data S3. *F* and *p* values obtained from post hoc ANOVA comparisons for the data presented in Figure 3.

Fig. 4b	VDAC1		
ANOVA	F	*p*	
	12,54	0,001*	
post-hoc	Ctr	ATOR	SIM
Ctr		0.017975	0.001086
ATOR	0.017975		0.252084
SIM	0.001086	0.252084	

	PGC1a		
ANOVA	F	*p*	
	15,65	,000*	
post-hoc	Ctr	ATOR	SIM
Ctr		0.024231	0.000481
ATOR	0.024231		0.066386
SIM	0.000481	0.066386	

	TFAM		
ANOVA	F	*p*	
	6,859	,010*	
post-hoc	Ctr	ATOR	SIM
Ctr		0.625795	0.010041
ATOR	0.625795		0.053421
SIM	0.010041	0.053421	

	MFF		
ANOVA	F	*p*	
	10,33	,002*	
post-hoc	Ctr	ATOR	SIM
Ctr		0.002035	0.044980
ATOR	0.002035		0.216901
SIM	0.044980	0.216901	

Fig. 4d	pDRP1		
ANOVA	F	*p*	
	56,08	,000*	
post-hoc	Ctr	ATOR	SIM
Ctr		0.001375	0.000178
ATOR	0.001375		0.000220
SIM	0.000178	0.000220	

	DRP1		
ANOVA	F	*p*	
	,328	,725	
post-hoc	Ctr	ATOR	SIM
Ctr		0.989858	0.732731
ATOR	0.989858		0.810049
SIM	0.732731	0.810049	

	ratio pDRP1/DRP1		
ANOVA	F	*p*	
	17,83	,000*	
post-hoc	Ctr	ATOR	SIM
Ctr		0,028101	0,000232
ATOR	0,028101		0,050066
SIM	0,000232	0,050066	

	pERK1/2		
ANOVA	F	*p*	
	9,679	,003*	
post-hoc	Ctr	ATOR	SIM
Ctr		0.002451	0.111855
ATOR	0.002451		0.111530
SIM	0.111855	0.111530	

	ERK1/2		
ANOVA	F	*p*	
	2,462	,127	
post-hoc	Ctr	ATOR	SIM
Ctr		0.228157	0.141017
ATOR	0.228157		0.949079
SIM	0.141017	0.949079	

	ratio pERK1/2/ERK1/2		
ANOVA	F	*p*	
	9,239	,004*	
post-hoc	Ctr	ATOR	SIM
Ctr		0.003664	0.026396
ATOR	0.003664		0.517242
SIM	0.026396	0.517242	

Data S4. *F* and *p* values obtained from post hoc ANOVA comparisons for the data presented in Figure 4.

**Fig. 5a**	**glucose**					**pyruvate**					**glutamine**					**mixture**		
ANOVA	F	*p*			ANOVA	F	*p*			ANOVA	F	*p*			ANOVA	F	*p*	
	91,58	,000*				,879	,435				1,408	,272				1,703	,216	
post-hoc	Ctr	ATOR	SIM			Ctr	ATOR	SIM			Ctr	ATOR	SIM			Ctr	ATOR	SIM
Ctr		0,000178	0,000178		post-hoc		0,406498	0,724188		post-hoc		0,299772	0,964135		post-hoc		0,199226	0,468495
ATOR	0,000178		0,379872		ATOR	0,406498		0,850284		ATOR	0,299772		0,398530		ATOR	0,199226		0,818200
SIM	0,000178	0,379872			SIM	0,724188	0,850284			SIM	0,964135	0,398530			SIM	0,468495	0,818200	
**Fig. 5b**	**glucose**					**pyruvate**					**glutamine**					**mixture**		
ANOVA	F	*p*			ANOVA	F	*p*			ANOVA	F	*p*			ANOVA	F	*p*	
	57,70	,000*				7,318	,006*				2,689	,100				1,066	,369	
post-hoc	Ctr	ATOR	SIM			Ctr	ATOR	SIM			Ctr	ATOR	SIM			Ctr	ATOR	SIM
Ctr		0,000178	0,000178		post-hoc		0.009458	0.017127		post-hoc		0,157541	0,133217		post-hoc		0,947339	0,541717
ATOR	0,000178		0,860989		ATOR	0.009458		0.951617		ATOR	0,157541		0,994663		ATOR	0,947339		0,369831
SIM	0,000178	0,860989			SIM	0.017127	0.951617			SIM	0,133217	0,994663			SIM	0,541717	0,369831	
**Fig. 5c**	**glucose**					**pyruvate**					**glutamine**					**mixture**		
ANOVA	F	*p*			ANOVA	F	*p*			ANOVA	F	*p*			ANOVA	F	*p*	
	5,853	,013*				26,28	,000*				51,82	,000*				152,0	,000*	
post-hoc	Ctr	ATOR	SIM		post-hoc	Ctr	ATOR	SIM		post-hoc	Ctr	ATOR	SIM		post-hoc	Ctr	ATOR	SIM
Ctr		0,039557	0,017003		Ctr		0.000183	0.000716		Ctr		0,000178	0,000179		Ctr		0,000178	0,000178
ATOR	0,039557		0,902031		ATOR	0.000183		0.096442		ATOR	0,000178		0,178973		ATOR	0,000178		0,482401
SIM	0,017003	0,902031			SIM	0.000716	0.096442			SIM	0,000179	0,178973			SIM	0,000178	0,482401	
**Fig. 5d**	**glucose**					**pyruvate**					**glutamine**					**mixture**		
ANOVA	F	*p*			ANOVA	F	*p*			ANOVA	F	*p*			ANOVA	F	*p*	
	18,81	,000*				11,11	,001*				,235	,793				5,629	,015*	
post-hoc	Ctr	ATOR	SIM		post-hoc	Ctr	ATOR	SIM		post-hoc	Ctr	ATOR	SIM		post-hoc	Ctr	ATOR	SIM
Ctr		0,000378	0,000398		Ctr					Ctr		0,929765	0,775461		Ctr		0,018680	0,045552
ATOR	0,000378		0,998869		ATOR					ATOR	0,929765		0,945121		ATOR	0,018680		0,890758
SIM	0,000398	0,998869			SIM					SIM	0,775461	0,945121			SIM	0,045552	0,890758	

Data S5. F and p values obtained from post hoc ANOVA comparisons for the data presented in Figure 5.

**Fig. 6a**	**malate+pyruvate**					**succinate+rotenone**					**RCRoligo**		
ANOVA	F	*p*			ANOVA	F	*p*			ANOVA	F	*p*	
	8,016	,004*				,926	,418				,615	,553	
post-hoc	Ctr	ATOR	SIM		post-hoc	Ctr	ATOR	SIM		post-hoc	Ctr	ATOR	SIM
Ctr		0,004083	0,034360		Ctr		0,730956	0,387164		Ctr		0,928624	0,534362
ATOR	0,004083		0,537514		ATOR	0,730956		0,825063		ATOR	0,928624		0,754215
SIM	0,034360	0,537514			SIM	0,387164	0,825063			SIM	0,534362	0,754215	
**Fig. 6b**	**malate+pyruvate**					**succinate+rotenone**					**RCRoligo**		
ANOVA	F	*p*			ANOVA	F	*p*			ANOVA	F	*p*	
	,351	,710				9,677	,002*				14,20	,000*	
post-hoc	Ctr	ATOR	SIM		post-hoc	Ctr	ATOR	SIM		post-hoc	Ctr	ATOR	SIM
Ctr		0.868505	0.690158		Ctr		0.014351	0.002238		Ctr		0.001751	0.000711
ATOR	0.868505		0.944492		ATOR	0.014351		0.615942		ATOR	0.001751		0.859096
SIM	0.690158	0.944492			SIM	0.002238	0.615942			SIM	0.000711	0.859096	
**Fig. 6c**	**malate+pyruvate**					**succinate+rotenone**					**RCRoligo**		
ANOVA	F	*p*			ANOVA	F	*p*			ANOVA	F	*p*	
	6,494	,009*				49,74	,000*				9,758	,002*	
post-hoc	Ctr	ATOR	SIM		post-hoc	Ctr	ATOR	SIM		post-hoc	Ctr	ATOR	SIM
Ctr		0,011250	0,034182		Ctr		0,000180	0,000178		Ctr		0.011232	0.002393
ATOR	0,011250		0,839016		ATOR	0,000180		0,140595		ATOR	0.011232		0.712654
SIM	0,034182	0,839016			SIM	0,000178	0,140595			SIM	0.002393	0.712654	

Data S6. *F* and *p* values obtained from post hoc ANOVA comparisons for the data presented in Figure 6.

Fig. 7a	CoQ9red			
ANOVA	F	*p*		
	222,3	,000*		
post-hoc	Ctr	Q10	SIM	Q10+SIM
Ctr		0,000879	0,000175	0,000175
Q10	0,000879		0,000175	0,000175
SIM	0,000175	0,000175		0,001928
SIM+Q10	0,000175	0,000175	0,001928	

	CoQ9ox			
ANOVA	F	*p*		
	56,90	,000*		
post-hoc	Ctr	Q10	SIM	Q10+SIM
Ctr		0.000180	0.001706	0.000189
Q10	0.000180		0.000175	0.965763
SIM	0.001706	0.000175		0.000175
SIM+Q10	0.000189	0.965763	0.000175	

	CoQ9redox			
ANOVA	F	*p*		
	475,0	0,00*		
post-hoc	Ctr	Q10	SIM	Q10+SIM
Ctr		0.001169	0.000175	0.000175
Q10	0.001169		0.000175	0.000175
SIM	0.000175	0.000175		0.063845
SIM+Q10	0.000175	0.000175	0.063845	

Fig. 7b	CoQ10red			
ANOVA	F	*p*		
	384.6	,000*		
post-hoc	Ctr	Q10	SIM	Q10+SIM
Ctr		0,000001	0,000001	0,000001
Q10	0,000001		0,000001	0,000001
SIM	0,000001	0,000001		0,000001
SIM+Q10	0,000001	0,000001	0,000001	

	CoQ10ox			
ANOVA	F	*p*		
	736	,000*		
post-hoc	Ctr	Q10	SIM	Q10+SIM
Ctr		0,000001	0,000001	0,000001
Q10	0,000001		0,000001	0,550000
SIM	0,000001	0,000001		0,000001
SIM+Q10	0,000001	0,550000	0,000001	

	CoQ10redox			
ANOVA	F	*p*		
	127,8	,000*		
post-hoc	Ctr	Q10	SIM	Q10+SIM
Ctr		0,000175	0,000226	0,000175
Q10	0,000175		0,000175	0,005776
SIM	0,000226	0,000175		0,000175
SIM+Q10	0,000175	0,005776	0,000175	

Fig. 7c				
ANOVA	F	*p*		
	1020	,000*		
post-hoc	Ctr	Q10	SIM	Q10+SIM
Ctr		0,000175	0,000175	0,000175
Q10	0,000175		0,000175	0,000240
SIM	0,000175	0,000175		0,000175
SIM+Q10	0,000175	0,000240	0,000175	

Fig. 7d				
ANOVA	F	*p*		
	78,36	,000*		
post-hoc	Ctr	Q10	SIM	Q10+SIM
Ctr		0.000175	0.000175	0.000175
Q10	0.000175		0.001587	0.386081
SIM	0.000175	0.001587		0.053284
SIM+Q10	0.000175	0.386081	0.053284	

Fig. 7e				
ANOVA	F	*p*		
	42,44	,000*		
post-hoc	Ctr	Q10	SIM	Q10+SIM
Ctr		0.000175	0.000180	0.000175
Q10	0.000175		0.003616	0.121327
SIM	0.000180	0.003616		0.365344
SIM+Q10	0.000175	0.121327	0.365344	

Fig. 7f				
ANOVA	F	*p*		
	35,52	,000*		
post-hoc	Ctr	Q10	SIM	Q10+SIM
Ctr		0,001181	0,000327	0,179437
Q10	0,001181		0,000175	0,106376
SIM	0,000327	0,000175		0,000176
SIM+Q10	0,179437	0,106376	0,000176	

Data S7. F and p values obtained from post hoc ANOVA comparisons for the data presented in Figure 7.
