# Supplementary material for: Statin-Induced Coenzyme Q Deficiency Induces Metabolic Reprogramming in Astrocytes
Source: Antioxidants (Basel). 2026 Jun 7;15(6):725. doi: 10.3390/antiox15060725 (PMC13295249; doi:10.3390/antiox15060725)
Supplement: Supplementary file 1 [file antioxidants-15-00725-s001.zip › Supplementary Figures.pdf]

Supplementary Figures Wojcicki et al.

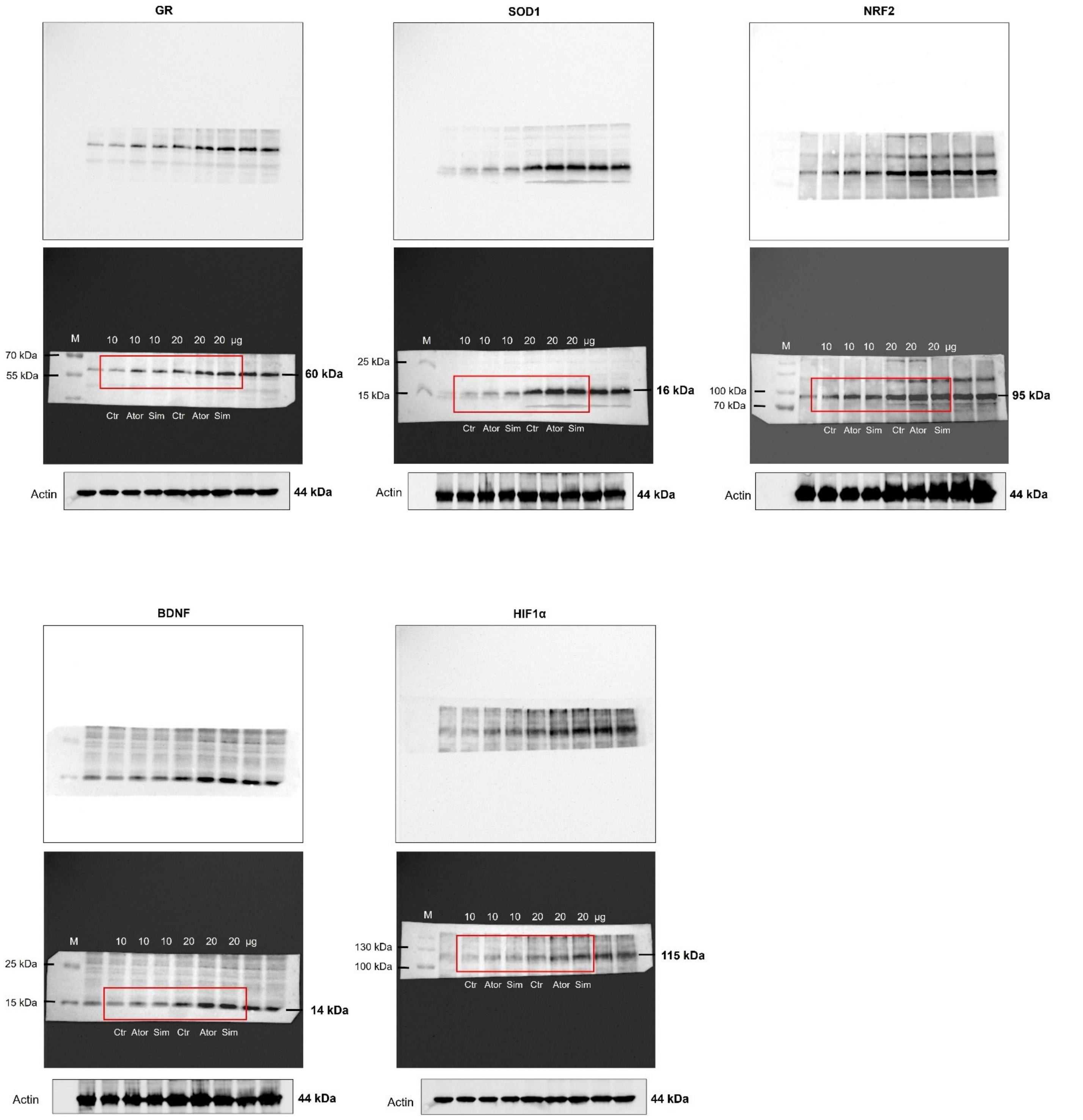

Figure S1. Uncropped images used to prepare Figure 2. Abbreviations: Ctr, control cells; Ator, atorvastatin-treated cells; Sim, simvastatin-treated cells; BDNF, brain-derived neurotrophic factor; GR, glutathione reductase; HIF1α, hypoxia-inducible factor 1α; NRF2, nuclear factor erythroid 2-related factor 2/NFE2-like bZIP transcription factor 2; SOD1, superoxide dismutase 1; M, molecular mass markers

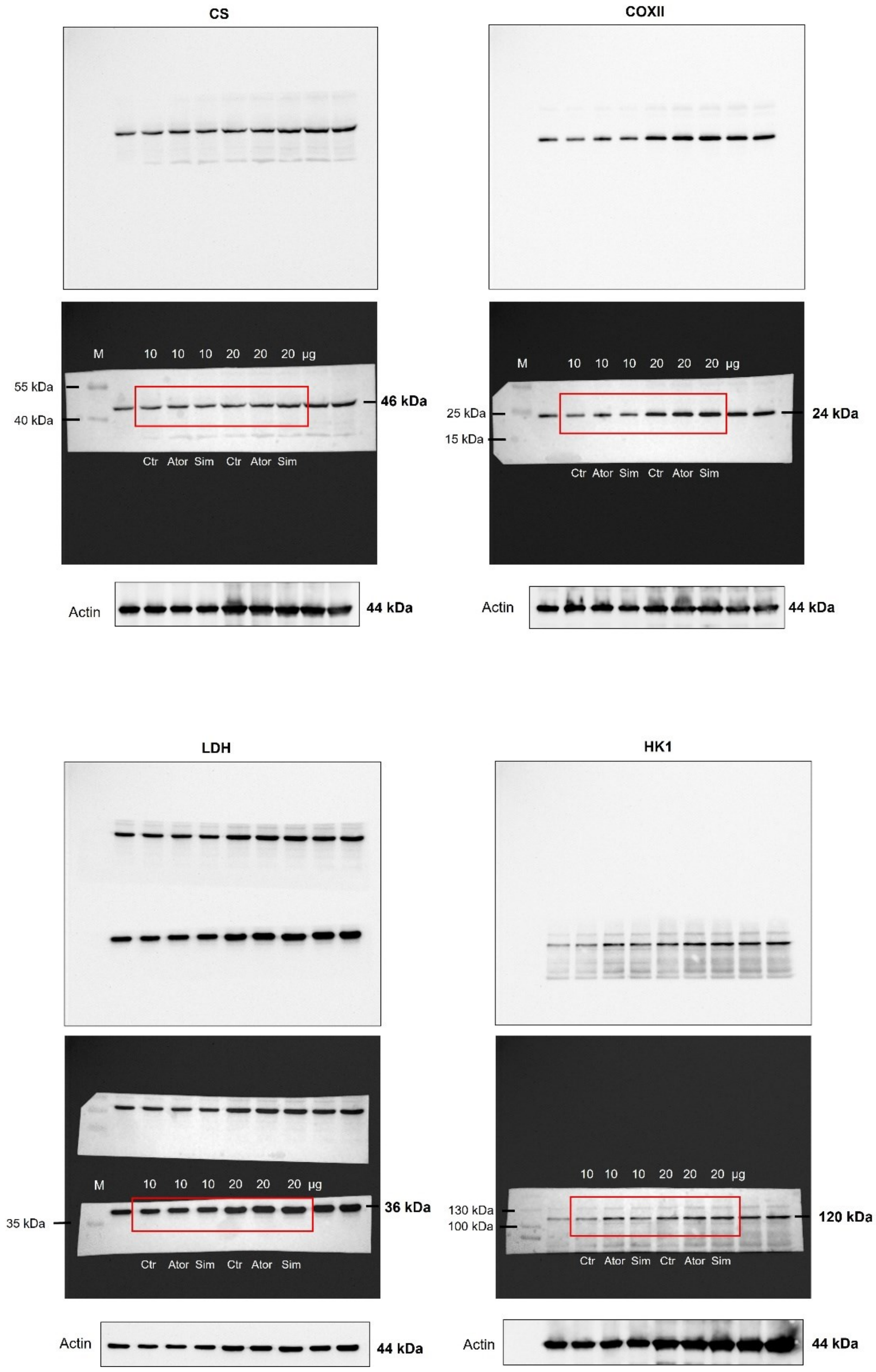

Figure S2. Original images used to prepare Figure 3. Abbreviations: Ctr, control cells; Ator, atorvastatin-treated cells; Sim, simvastatin-treated cells; COXII, cytochrome *c* oxidase subunit II; CS, citric synthase; HK1, hexokinase 1; LDH, lactate dehydrogenase; M, molecular mass markers

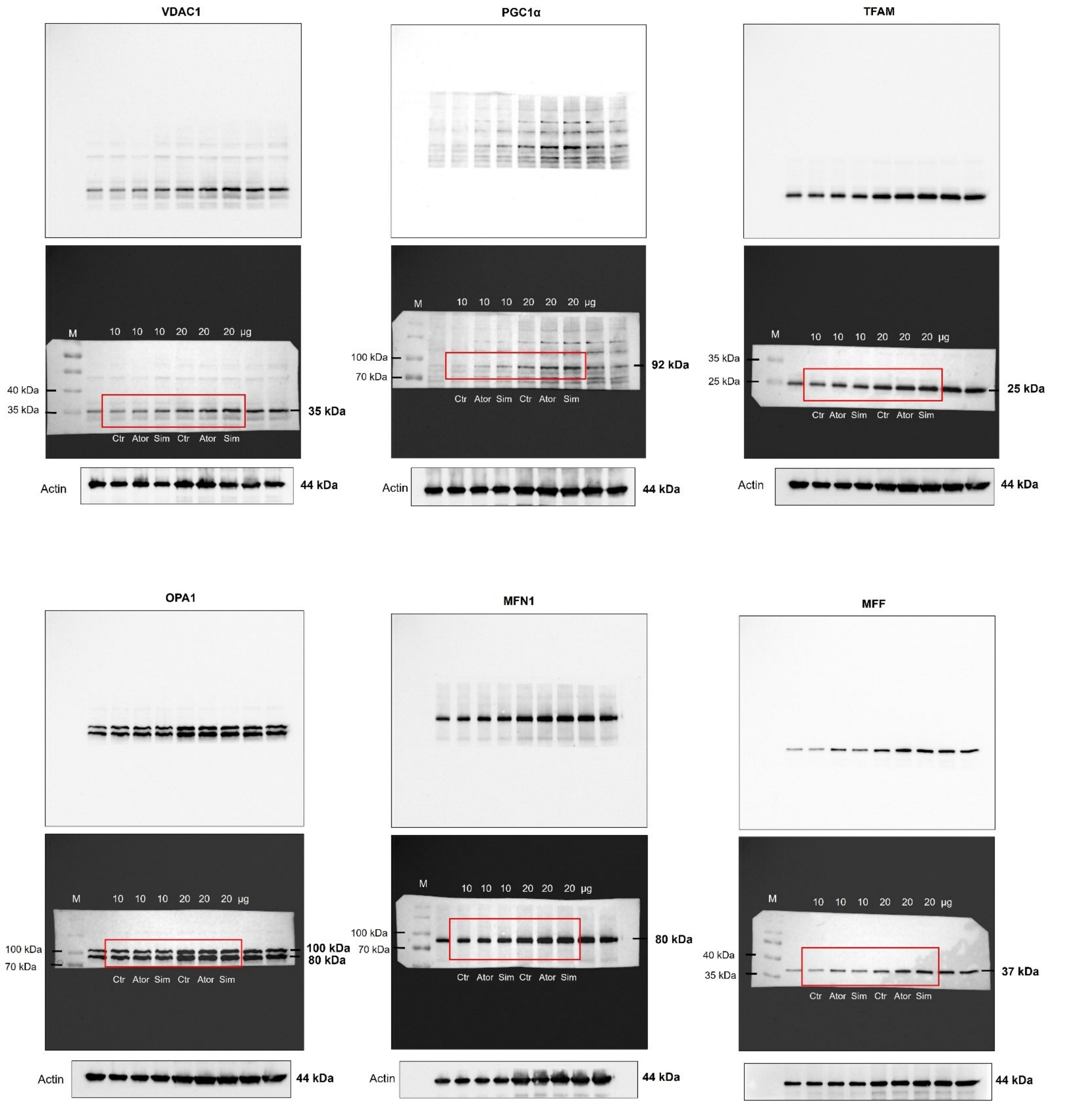

Figure S3. Original images used to prepare Figure 4a. Abbreviations: Ctr, control cells; Ator, atorvastatin-treated cells; Sim, simvastatin-treated cells; MFN1/2, mitofusin 1/2; MFF, mitochondrial fission factor; OPA1, OPA1 mitochondrial dynamin-like GTPase; PGC1α, peroxisome proliferator-activated receptor γ coactivator 1α; TFAM, transcription factor A, mitochondrial; VDAC1, voltage-dependent anion-selective channel 1; M, molecular mass markers

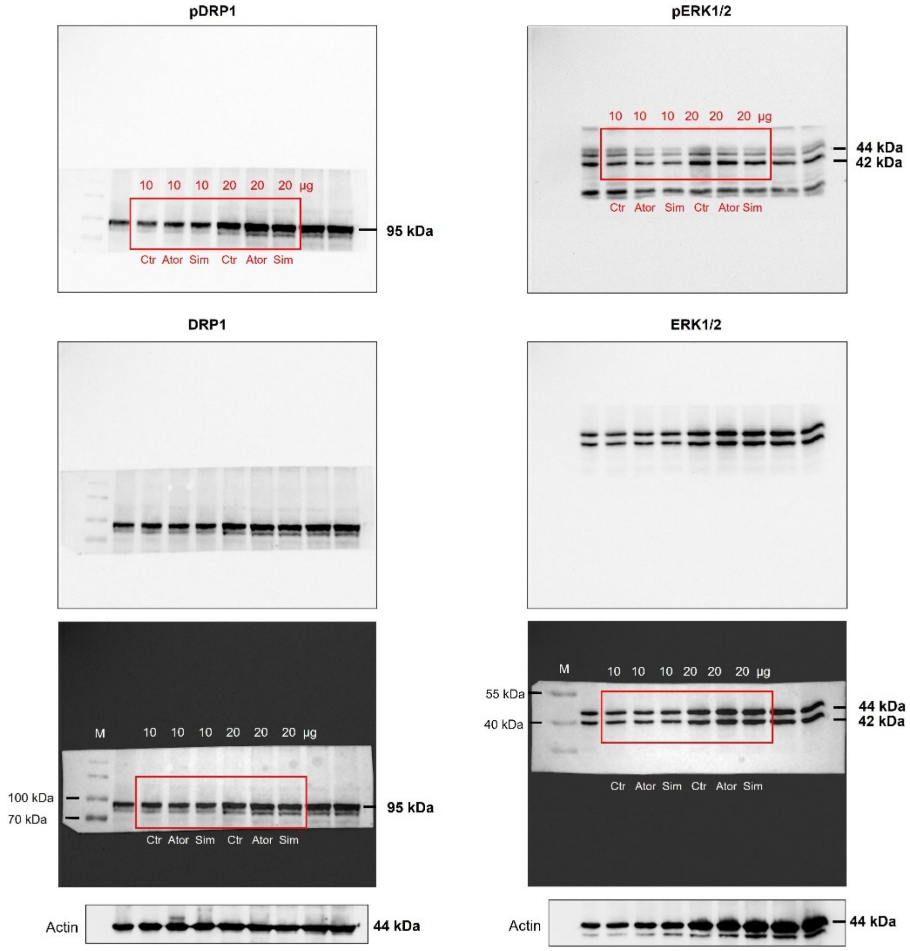

Figure S4. Original images used to prepare Figure 4b. Abbreviations: Ctr, control cells; Ator, atorvastatin-treated cells; Sim, simvastatin-treated cells; DRP1, total dynamin related protein 1; pDRP1, phosphorylated DRP1; ERK1/2, total extracellular signal-regulated protein kinases 1/2; p-ERK1/2, phosphorylated ERK1/2; M, molecular mass markers

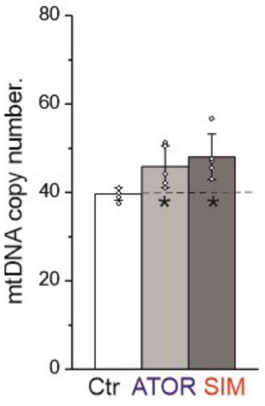

Figure S5. Effect of 200 nM atorvastatin and simvastatin on mtDNA copy number. Mean ± SD, n = 5; statistics: KW ANOVA. Significance: *, $p < 0.05$ vs. control cells (Ctr). Abbreviations: ATOR, atorvastatin; mtDNA, mitochondrial DNA; SIM, simvastatin

Methods: Total DNA was isolated with QIAamp DNA Micro Kit (Qiagen) using protocol provided by manufacturer. Relative mitochondrial copy number analysis was performed using SsoAdvanced Universal SYBR Green Supermix (Bio-Rad) on a QuantStudio™ 7 Flex Real-Time PCR System. For mitochondrial genome *Mt-nd1* gene (Mitochondrially Encoded NADH:Ubiquinone Oxidoreductase Core Subunit 1, Gene ID: 26193, protein accession: YP_665629.1) was amplified, for nuclear genome *Beta-actin* gene was amplified. Starters for *Mt-nd1* were designed by BLAST (https://blast.ncbi.nlm.nih.gov/Blast.cgi), primers for *Beta-actin* (GenBank accession number NM_031144) was described previously [1]. Reactions were carried out in 10 µl volume containing: 2 ng isolated DNA, 5 µl of Supermix, 350 nm of primers for *Mt-nd1* or 500 nm of primers for *Beta-actin* respectively and water up to 10 µl. Reactions conditions was: initial denaturation 98°C for 5 minutes followed by 40 cycles of denaturation at 95°C for 15 s and annealing at 62°C for 1 minute. Relative mitochondria copy number was calculated using formula: 2 × 2ΔCT, ΔCT = (nucDNA CT – mtDNA CT) as described previously [2].

MT-ND1

F: 5' CCTAGGCCCCTTATATCACATCA

R: 5' TCGAAAACGGGGGTAGGATG

Beta-actin

F: 5'GGGATGTTTGCTCCAACCAA

R: 5'GCGCTTTTGACTCAAGGATTTAA

References

1. Nicklas, J.A.; Brooks, E.M.; Hunter, T.C.; Single, R.; Branda, R.F. Development of a quantitative PCR (TaqMan) assay for relative mitochondrial DNA copy number and the common mitochondrial DNA deletion in the rat. *Environ. Mol. Mutagen.* **2004**, *44*, 313–320, doi:10.1002/em.20050.
2. Rooney, J.P.; Ryde, I.T.; Sanders, L.H.; Howlett, E.H.; Colton, M.D.; Germ, K.E.; Mayer, G.D.; Greenamyre, J.T.; Meyer, J.N. PCR based determination of mitochondrial DNA copy number in multiple species. *Methods Mol. Biol.* **2015**, *1241*, 23–38, doi:10.1007/978-1-4939-1875-1_3.
